# Quality and quantity control of proteins in senescence

**DOI:** 10.18632/aging.100145

**Published:** 2010-05-18

**Authors:** Masashi Narita

**Affiliations:** Cancer Research UK, Cambridge Research Institute, Li Ka Shing Centre, Cambridge CB2 0RE, United Kingdom

**Keywords:** autophagy, senescence, oncogene, aging

## Abstract

Autophagy has been implicated in aging and age-related diseases but its roles in these processes are far from straightforward: the anti-aging effect of autophagy has been shown in lower eukaryotes, and both pro- and anti-tumorigenic effects of autophagy have also been demonstrated. The new link between autophagy and senescence provides an insight into the diversified downstream effects of autophagy in various cellular contexts.

Macroautophagy (referred to as autophagy hereafter) is a highly conserved lysosome-mediated catabolic process, which can deal with the bulk degradation of cytoplasmic proteins as well as small organelles. Although the activation of autophagy can be acutely induced by nutrient deprivation, it is also known that cells exhibit a basal level of autophagy activity. Thus, autophagy plays important roles in the fine-tuning of energy homeostasis and the quality control of proteins and small organelles [1,2].

In addition to metabolic stress, it has been shown that autophagy can also be induced by various cytotoxic stresses. Not surprisingly, increasing evidence has shown that autophagy is involved in a number of pathophysiologies, including aging and age-related diseases (cancer, atherosclerosis, and neuro-degeneration), and innate and adaptive immunity [3]. It is still not entirely clear, however, how such a catabolic program contributes to the cytotoxic stress response. Since autophagy is thought to be a survival as well as a non-apoptotic cell death mechanism, it could be an effector for or against stress responsive phenotypes depending on the context [4,5].

## Replicative senescence (RS) and oncogene-induced senescence (OIS)

Cellular senescence was originally defined as ‘irreversible' cell cycle arrest caused by replicative exhaustion in cultured human diploid fibroblasts (HDFs) [6]. Later, it was shown that this ‘replicative exhaustion' is essentially telomere shortening, which activates a persistent DNA damage response [7]. The senescence trigger is, however, not restricted to telomere dysfunction. In 1997, Serrano *et al.* showed that oncogenic Ras, which can transform immortalized cells, induces a senescence-like phenotype in normal HDFs [8]. This is rather paradoxical, but it was shown that the initial response of cells to oncogenic Ras is hyper-proliferation. Thus, it was proposed that cells somehow sense this abnormal proliferation, and undergo senescence as a delayed response to counter the oncogenic signals [9]. It is conceivable that these 'delayed responses' would include effector mechanisms of senescence, and understanding these mechanisms would provide insights into senescence-associated pathophysiologies, including aging and cancer. Indeed, OIS in culture has been a very useful system for the identification and characterization of senescence effector mechanisms, such as epigenetic gene regulation and chromatin modifications, DNA damage response, negative feedback in the PI3K pathway, and senescence-associated secretory phenotype (SASP)/senescence-mess secretome (SMS) [10-14]. Our recent study has added autophagy to the list of OIS effector mechanisms [15].

Irrespective of the triggers, senescence shares many, if not all, of the effector mechanisms identified in OIS systems to some extent. Therefore it is not surprising that autophagy is also implicated in RS [16]. However, despite the similarity of the endpoint between RS and OIS, the modes of senescence establishment are distinct: RS involves modest but long-term exposure of cells to stress and HDFs reach a senescent state over several months, while OIS establishment is a more acute and dynamic process. It remains to be addressed how these distinct conditions share the regulatory mechanisms of autophagy and its downstream effects.

Based on the intensity of the stress and acuteness of the process, RS and OIS may reflect natural aging and age-related disease (e.g. cancer and atherosclerosis), respectively. Interestingly, many senescence effector mechanisms, including autophagy, have also been implicated in both aging and age-related disease [3,17-20]. Autophagy in lower eukaryotes has been shown to be critical for the anti-aging effects of dietary restriction and negative modulation of insulin-signalling [21-24]. In contrast to its anti-aging effect, as shown in various models, autophagy can have either pro- or anti-tumorigenic activity depending on the context [3,20]. Thus it is possible that the same cellular machinery plays distinct roles ageing and age-related diseases.

In RS, Gamerdinger et al. (2009) showed that there is a gradual shift from the proteasome pathway to autophagy within polyubiquitinated protein degradation systems. This shift is mediated through at least two members of the BAG (Bcl-2-associated athanogene) protein family, which can bind to chaperones of the Hsc/HSP70 family and thereby modulate protein quality control. They showed that BAG1 and BAG3 positively regulate the proteasomal and autophagic pathways, respectively, and that BAG1 and BAG3 levels are reciprocally regulated during RS, in which the BAG3/BAG1 ratio is elevated [16].

The increase of BAG3/BAG1 ratio and activation of autophagy is also found in tissue aging, thus, it is not limited to *in vitro* "cell aging". Gamerdinger et al. (2009) found a similar age-related correlation between autophagy and the BAG3/BAG1 ratio in rodent brains. Considering the age-dependent accumulation of damaged proteins (particularly due to oxidative stress), the role of autophagy in this case may be classic ‘quality control' of proteins and other macromolecules. This is also consistent with the anti-aging role of autophagy as described earlier. However, it has also been noted that global autophagy capacity declines with age *in vivo* [25,26]. How can one reconcile the apparent discrepancy? First, it is possible that the extent to which autophagy activity changes is different depending on cell type. It has been demonstrated in aged brains that neurons, but not astrocytes, show upregulated autophagy [16]. Second, it is also possible that it is the basal activity and metabolic regulation of autophagy that decline during aging, but cytotoxic stress-induced autophagy may not be severely affected particularly in long-lived cells, which are susceptible to the accumulation of oxidative stress. Interestingly, it was recently reported that progeroid mouse models exhibit an extensive activation of the basal autophagy [27]. It still remains to be elucidated, however, whether the chronic activation of autophagy in these mice is a protective reaction against the causal elements associated with premature aging symptoms or that autophagy actively contributes to the phenotype. This study, in conjunction with the observations by Gamerdinger et al. (2009), suggests that alteration of autophagy activity during aging and the functional implications of autophagy in age-associated pathophysiologies can be more complex, at least in mammals.

**Figure 1.**
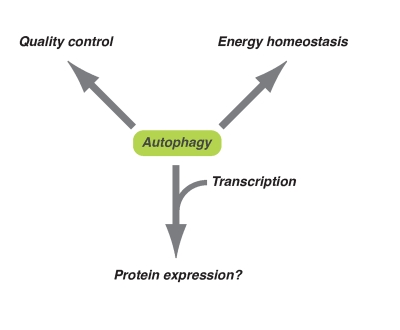
Diversified downstream effects of autophagy. Autophagy plays an important role in energy homeostasis and quality control of macromolecules at the basal level or occurs during long-term exposure to oxidative stress. On the other hand, in response to acute cytotoxic stresses (e.g. oncogenic stress), autophagy might contribute to the expression of some proteins together with epigenetic transcriptional regulation.

## From transcription to proteins

If autophagy is involved in the long-term quality control of cytoplasmic macromolecules, as proposed in Gamerdinger (2009), what is the acute role of autophagy during OIS? To ask this question, we have focused on its highly dynamic nature. This is more obvious when inducible oncogenes are used, such as 4-hydroxytamoxifen (4-OHT)-inducible ER:Ras fusion protein, which is comprised of a mutant form of the estrogen receptor ligand-binding domain and constitutively active H-RasV12 [15]. This inducible system allows us to focus on the transition phase, which lies between the initial mitotic burst after Ras-induction, and the static senescence phase. It is possible that the most dramatic phenotypic remodelling and cellular adjustments to the new environment occur during this transition phase.

One obvious mechanism that is responsible for this transition is a global transcription change. We and others previously described a unique chromatin structure - senescence associated heterochromatic foci (SAHFs) - which seem to play a role in transcriptional regulation during senescence [10,28,29]. Indeed, many senescence-associated genes are upregulated during the transition phase, including a large number of secretory proteins. Among these, IL6 and IL8, an inflammatory cytokine and chemokine respectively, have recently been shown to reinforce the senescence phenotype, thus representing another senescence effector mechanism - SASP/SMS [30-32]. The timing of IL6/8 induction has been correlated with autophagy activation during the transition phase. Strikingly, RNAi-mediated repression of *Atg5* or *Atg7* (essential genes for autophagy) suppresses IL6/8 production, indicating a functional relevance of autophagy in senescence. Although it is still unclear exactly how autophagy facilitates IL6/8 production, we have shown that the transcription levels of these genes are often even higher when *Atg5* or *Atg7* are knocked-down, indicating that the positive regulation of these genes by autophagy occurs at the post-transcriptional level. Thus IL6/8, which are acutely produced *en masse*, seem to be regulated in a cooperative manner by mRNA and protein synthesis (Figure 1). Massive induction of autophagy and the resultant efficient protein turnover might provide another layer of gene expression control - at least for some genes - to execute epigenetic 'blueprints' during OIS.

## Perspective

Metabolism is a very dynamic and robust process, thus interpreting 'snapshots' of metabolic processes can be difficult. Our recent study focusing on the dynamic phase of OIS highlighted the distinct role of autophagy in controlling protein quantity in OIS. Extensive characterization of autophagy's distinct and shared roles in RS and OIS would be beneficial to further understand the mechanisms by which autophagy has diverse effects in different contexts. In addition to its downstream effects, it is also important to understand how autophagy is regulated during senescence. Consistent with a previous report [12], we have shown that components of the PI3K pathway - including mTOR, a negative regulator of autophagy - are attenuated after their acute activation following Ras expression during the transition phase of OIS [15,33]. Although the long-term fluctuation of mTOR activity during the senescence phase remains to be fully characterized, our study raises an interesting question: how protein synthesis (positively regulated by mTOR) and autophagy (negatively regulated by mTOR) are activated during the senescence transition. Interestingly, recent reports show that mTOR inhibition by rapamycin decelerates senescence [34,35]. mTOR-regulated catabolic and anabolic processes seems to be somehow coupled to contribute to senescence, and perhaps aging.

## Acknowledgments

MN is supported by the University of Cambridge, Cancer Research UK and Hutchison Whampoa Limited. We thank Masako Narita for critical reading of the manuscript and Laura Blackburn for editing.
